# A unifying conceptual model for the environmental responses of isoprene emissions from plants

**DOI:** 10.1093/aob/mct206

**Published:** 2013-09-19

**Authors:** Catherine Morfopoulos, Iain C. Prentice, Trevor F. Keenan, Pierre Friedlingstein, Belinda E. Medlyn, Josep Peñuelas, Malcolm Possell

**Affiliations:** 1 Department of Life Sciences, Imperial College, Silwood Park, Ascot SL5 7PY, UK; 2 AXA Chair of Biosphere and Climate Impacts, Department of Life Sciences and Grantham Institute for Climate Change, Imperial College, Silwood Park, Ascot SL5 7PY, UK; 3 Department of Biological Sciences, Macquarie University, Sydney, NSW 2109, Australia; 4 College of Engineering, Mathematics and Physical Sciences, Streatham Campus, University of Exeter, Exeter, EX4 4QF, UK; 5 CREAF, Cerdanyola del Vallés E-,08193, Barcelona, Spain; 6 CSIC, Global Ecology Unit CREAF-CEAB-UAB, Cerdanyola del Vallés, 08193, Barcelona, Spain; 7 Faculty of Agriculture and Environment, The University of Sydney, Sydney, NSW 2006, Australia

**Keywords:** Isoprene, modelling, electron transport, photosynthesis, temperature, carbon dioxide, isoprene emission, volatile organic compounds

## Abstract

**Background and Aims:**

Isoprene is the most important volatile organic compound emitted by land plants in terms of abundance and environmental effects. Controls on isoprene emission rates include light, temperature, water supply and CO_2_ concentration. A need to quantify these controls has long been recognized. There are already models that give realistic results, but they are complex, highly empirical and require separate responses to different drivers. This study sets out to find a simpler, unifying principle.

**Methods:**

A simple model is presented based on the idea of balancing demands for reducing power (derived from photosynthetic electron transport) in primary metabolism versus the secondary pathway that leads to the synthesis of isoprene. This model's ability to account for key features in a variety of experimental data sets is assessed.

**Key results:**

The model simultaneously predicts the fundamental responses observed in short-term experiments, namely: (1) the decoupling between carbon assimilation and isoprene emission; (2) a continued increase in isoprene emission with photosynthetically active radiation (PAR) at high PAR, after carbon assimilation has saturated; (3) a maximum of isoprene emission at low internal CO_2_ concentration (*c*_i_) and an asymptotic decline thereafter with increasing *c*_i_; (4) maintenance of high isoprene emissions when carbon assimilation is restricted by drought; and (5) a temperature optimum higher than that of photosynthesis, but lower than that of isoprene synthase activity.

**Conclusions:**

A simple model was used to test the hypothesis that reducing power available to the synthesis pathway for isoprene varies according to the extent to which the needs of carbon assimilation are satisfied. Despite its simplicity the model explains much in terms of the observed response of isoprene to external drivers as well as the observed decoupling between carbon assimilation and isoprene emission. The concept has the potential to improve global-scale modelling of vegetation isoprene emission.

## INTRODUCTION

Isoprene (2-methyl-1,3-butadiene; C_5_H_8_) is a highly volatile and reactive unsaturated hydrocarbon that is produced continuously in daylight by many terrestrial plants, and in great abundance by broadleaved trees. On a mass basis, it is the most important biogenic volatile organic compound (BVOC) emitted by vegetation, with an annual global emission of approximately 0·5 × 10^15^ g C. This is similar in magnitude to the total annual emission of the greenhouse gas methane (CH_4_) from all natural sources combined (Guenther *et al.*, 1995, 2006; Laothawornkitkul *et al.*, 2009). Although not a greenhouse gas itself, isoprene reacts in the atmosphere with oxidants, including hydroxyl radicals (OH) and ozone (O_3_) (Fan and Zhang, 2004), and consequently influences the atmospheric lifetime and concentration of CH_4_ (Poisson *et al.*, 2000; Collins *et al.*, 2002, 2010; Pike and Young, 2009). The influence of isoprene on atmospheric oxidation capacity has been proposed as one of the controls of the glacial–interglacial variations of atmospheric CH_4_, as recorded in ice cores (Valdes *et al.*, 2005; Singarayer *et al.*, 2011). Isoprene also enhances the production of tropospheric ozone (O_3_), a potent greenhouse gas and toxic pollutant, under high-NO_*x*_ conditions (Sanderson *et al.*, 2003; Hauglustaine *et al.*, 2005), and can significantly affect the atmosphere's radiative balance through the generation of secondary organic aerosols (Claeys *et al.*, 2004; Heald *et al.*, 2008; Carlton *et al.*, 2009; Nozière *et al.*, 2011).

Isoprene emissions by plants at the leaf scale respond to changes in photosynthetically active radiation (PAR), temperature, ambient CO_2_ concentration and drought (Sharkey and Yeh, 2001; Laothawornkitkul *et al.*, 2009; Pacifico *et al.*, 2009; Niinemets, 2010). Despite general agreement between models under the present climate, simulations of future isoprene emissions, and their potential impact on atmospheric chemistry, change dramatically depending on the temperature and light responses of the model (Keenan *et al.*, 2009) and whether the model includes a physiological response of isoprene emission to CO_2_ (Heald *et al.*, 2009; Young *et al.*, 2009; Pacifico *et al.*, 2012). Given the continuously increasing atmospheric CO_2_ concentration and its impact on future temperature, we need to understand the processes behind observed responses, and use that understanding to build better models.

The adaptive significance of isoprene emission is thought to be connected with enhancing membrane stability at high temperatures, and protection against oxidative stress – including that induced by high temperatures (Sharkey and Yeh, 2001; Vickers *et al.*, 2009; Velikova *et al.*, 2011, 2012). On time scales of weeks to years, acclimation mechanisms acting at the level of gene transcription may operate, possibly in such a way as to match isoprene synthase activity to adaptive requirements (Grote and Niinemets, 2008; Monson *et al.*, 2012; Harrison *et al.*, 2013). Here, however, we focus on the immediate responses of isoprene emission to environmental variations, as observed in experiments conducted over a time scale of minutes to hours, and the basic metabolic mechanisms that may be responsible for them.

The biosynthesis of isoprene occurs via the chloroplastic methylerythritol 4-phosphate (MEP) pathway (Lichtenthaler, 1999; Logan *et al.*, 2000; Sharkey *et al.*, 2008). ^13^C labelling experiments have shown that the majority of the C in isoprene comes directly from photosynthesis, with the remainder coming from cytosolic C pools depending upon the environmental conditions (Delwiche and Sharkey, 1993; Kreuzwieser *et al.*, 2002; Karl *et al.*, 2002; Affek and Yakir, 2003; Loreto *et al.*, 2004). However, the metabolic controls of the MEP pathway are only beginning to be elucidated (Li and Sharkey, 2012). With incomplete understanding of the metabolic controls of the pathway, models have been developed on the basis of experimental studies of the relationships between isoprene emission and environmental variables. The approach with the longest pedigree combines empirically derived functions for each environmental effect: this is the principle of the MEGAN model (Guenther *et al.*, 2006), developed from the pioneer work of Guenther *et al.* (1993). Other approaches have made more direct use of the limited available information at the biochemical process level, e.g. SIM-BIM (Zimmer *et al.*, 2000, 2003) and the models of Niinemets *et al.* (1999) and Martin *et al.* (2000). Aside from the model from Martin *et al.* (2000), which has an ATP limitation for isoprene production at high internal CO_2_ concentration (*c*_i_), all these models need an empirical parameterization to reproduce the observed CO_2_ response. This is potentially quite a severe limitation because there may be unforeseen interactions between the effects of different environmental drivers. Empirical models such as MEGAN include a multiplicity of functions for each environmental response of isoprene emission. More mechanistic approaches such as SIM-BIM, on the other hand, require information on many parameters. This might also be an issue because there is a generally accepted trade-off between the multiplicity of required parameter values and model robustness. We set out to identify a unifying principle that might transcend these limitations.

Our starting point was the model of Niinemets *et al.* (1999), which is based on quantifying the NADPH requirement of isoprene synthesis. Niinemets *et al.* (1999) assumed that a certain fraction of the total electron flux generated by Photosystem II is allocated to this function. The model we present here, initially proposed in Harrison *et al.* (2013), builds on Niinemets' work but differs in one fundamental respect: it links isoprene emission to the electron availability for isoprene emission, relative to the needs of CO_2_ assimilation. Therefore, the model predicts higher isoprene emissions when absorbed radiant energy (leading to the ‘supply’ of NADPH) exceeds the ‘demand’ for CO_2_ assimilation. An excess of energy arises because of a mismatch between light availability and carboxylation capacity, which typically occurs daily – especially at high PAR, associated high temperature and under water stress. We compare the model's predictions of observed, published environmental responses of isoprene emission to changes in PAR and the leaf-internal concentration of CO_2_ (*c*_i_) with those obtained with the Guenther *et al.* (1993) algorithm, hereafter called G93, which is the basis of the widely used MEGAN model (Guenther *et al.*, 2006), and with the model of Niinemets *et al.* (1999), hereafter called the Niinemets model. We also compare the theoretical temperature responses of our model with G93 and the Niinemets model. We focus on these two models as they have been widely used at the global scale (Guenther *et al.*, 2006; Lathière *et al.*, 2010; Arneth *et al.*, 2011; Pacifico *et al.*, 2012). However, other isoprene models have been developed. Reviews can be found in Arneth *et al.* (2007*a*), Grote and Niinemets (2008), and Monson *et al.* (2012).

## HYPOTHESIS

In isoprene-emitting plants with the C_3_ pathway of photosynthesis, over 90 % of isoprene production takes place in the chloroplast via the MEP pathway (Lichtenthaler *et al.*, 1997; Sharkey *et al.*, 2008). The final stage is the enzymatic synthesis of isoprene from its precursor, dimethylallyl diphosphate (DMADP). On a per-molecule basis, isoprene synthesis is energetically expensive, and has a high requirement for reducing power (14 NADPH for one molecule of isoprene). For comparison, only six NADPH are needed to synthesize glyceradehyde 3-phosphate (G3P), and only five for pyruvate. NADPH consumption for G3P and pyruvate synthesis takes place within the Calvin cycle and therefore is linked to the electron cost for carbon assimilation. Three additional reducing steps are needed within the MEP pathway to reduce G3P and pyruvate to DMADP. These supplementary reducing steps consume one further NADPH, and two additional reducing equivalents in the form of either NADPH or ferredoxin (Fd) (Charon *et al.*, 1999; Hecht *et al.*, 2001; Seemann *et al.*, 2006; Li and Sharkey, 2012). Our hypothesis focuses on these additional reduction steps, which are directly linked to the production of isoprene.

Isoprene production is typically measured in nanomoles per second while photosynthesis and respiration rates (to which G3P and pyruvate production are linked) are measured in micromoles per second. Hence, the major consumption of reducing power takes place within the Calvin cycle and associated photorespiration while the diversion of reducing power to the MEP pathway is very small. Yet there is abundant circumstantial evidence for a link between the availability of reducing power (after the requirements of carbon assimilation have been accounted for) and the magnitude of this diversion. The MEP pathway is tightly linked to the photosynthetic apparatus, involves light-dependent reactions and takes place in the chloroplast. Higher isoprene emission capacity is encountered under conditions when photoinhibition occurs, including high light intensities, low *c*_i_ and high temperatures. Isoprene emissions decrease if plants are fed with nitrate (note that nitrate reduction to ammonia occurs mainly in the cytoplasm and consumes NAPDH) instead of being fed with ammonia directly (Campbell, 1988; Rosenstiel *et al.*, 2004). Li and Sharkey (2012) measured an extremely high level of the intermediate metabolite, methylerythritol cyclodiphosphate (MEcDP), in an N_2_ atmosphere, where the carbon assimilation and photorespiration sinks for NADPH are blocked. Thus, it might be that the MEP pathway acts as a ‘branch circuit’ with the amount of NAPDH allocated to it increasing in proportion to the amount of reducing power to spare from other functions.

**Fig. 1.**
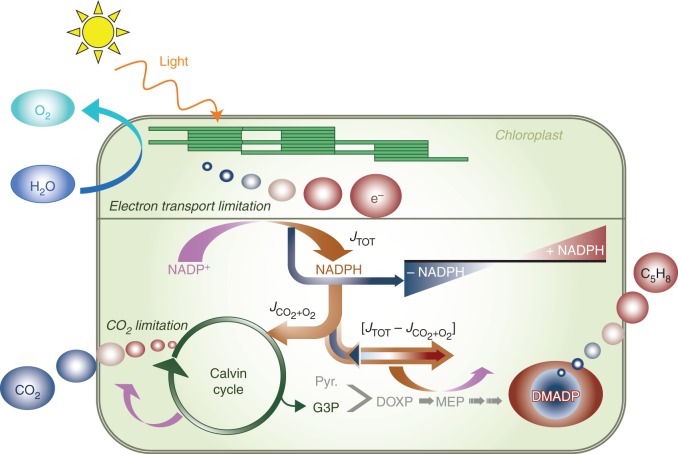
Schematic of the processes underlying the model of isoprene emissions. The availability of reducing power (NADPH) for CO_2_ assimilation is represented by a colour scheme, from dark blue (deficit of NADPH) to dark red (excess of NADPH). Symbols: NADPH and NADP^+^, nicotinamide adenine dinucleotide phosphate; DMADP, dimethylallyl diphosphate; Pyr, pyruvate; G3P, glyceraldehyde 3-phosphate; DOXP, 1-deoxy-d-xylulose 5-phosphate; MEP, 2-*C*-methyl-d-erythritol 4-phosphate.

Thus, we hypothesize that isoprene emission is regulated in the short term by variations of the DMADP pool size, linked to the excess or deficit of electrons (and so also reducing power) relative to the needs of carbon assimilation. Figure 1 provides a schematic of the processes involved. When the chloroplast is illuminated, light absorbed by the thylakoids generates the electron flux (*J*_tot_) that finally reduces NADP^+^ to NADPH. Most of the NADPH is used in the Calvin cycle for carbon fixation, but the total NADPH thus generated (≈ 0·5 *J*_tot_) exceeds the amount consumed in the Calvin Cycle (≈ 0·5 *J*_CO2+O2_). When assimilation is light-limited (at high *c*_i_ and/or low PAR) there is still some NADPH available for other functions, which include nitrate reduction (Canvin and Atkins, 1974; Niinemets, 2004; Eichelmann *et al.*, 2011) and DMADP synthesis. When assimilation is Rubisco-limited (at low *c*_i_ and/or high PAR) this excess of NADPH becomes larger, allowing more NADPH to be used in DMADP synthesis.

This reasoning suggests the following simple model:
(1)
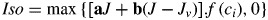

where *Iso* is the rate of isoprene emission; *f*(*c*_i_) is a function of internal CO_2_ concentration; *J* is an estimate of the total electron flux, taken to be a non-rectangular hyperbolic function of absorbed PAR and the maximum electron flux *J*_max_, following Farquhar *et al.* (1980); *J_v_* is the electron flux required to support Rubisco-limited carbon assimilation; and **a** and **b** are parameters. The electron flux required to support carbon assimilation is derived as follows (from Farquhar *et al.*, 1980):
(2)
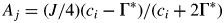

where *A*_j_ is the gross (light-limited) assimilation rate and Γ* is the CO_2_ compensation point in the absence of dark respiration. Hence
(3)
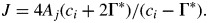



When Rubisco limits photosynthesis, then
(4)
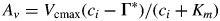

where *A*_v_ is the gross (Rubisco-limited) assimilation rate, *V*_cmax_ is the Rubisco capacity and *K*_m_ = *K*_c_(1 + [O_2_]/*K*_o_) where *K*_c_ and *K*_o_ are the Michaelis coefficients of Rubisco for CO_2_ and O_2_ respectively (Farquhar *et al.*, 1980). Substituting this into eqn (3) gives:
(5)
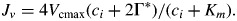

It should be noted that *J* in eqn (1) is used as an estimate of *J*_tot_ and could be an underestimate (Singsaas *et al.*, 2001; Niinemets, 2004). More details of the photosynthetic model, as used in this paper, can be found in the Supplementary Data.

The term **a***J* in eqn (1) represents a ‘baseline’ of isoprene emission under light-limited conditions under the equilibrium conditions for carbon assimilation (*J* = *J*_v_, energy supply = Rubisco demand), while **b**(*J* – *J*_v_) represents variation in isoprene emission due to the disequilibrium between supply and demand.

The function *f*(*c*_i_) in eqn (1) is chosen to take the value *c*_i_/Γ* when *c*_i_ ≤ Γ* and 1 otherwise. Because of this function, the model differs slightly from the one we proposed in Harrison *et al.* (2013). The function *f*(*c*_i_) reflects the idea that a minimum rate of supply of carbon chains is required for isoprene synthesis, and the common observation that isoprene emission ceases abruptly when *c*_i_ <Γ* (Wolfertz *et al.*, 2003; Rasulov *et al.*, 2009, 2011; Monson *et al.*, 2012; Sun *et al.*, 2012). This fall-off of isoprene at low *c*_i_ is not always observed: emission of isoprene in CO_2_-free air has been reported in a few studies (Monson and Fall, 1989; Affek and Yakir, 2003; Li and Sharkey, 2012), but comparable conditions are not found in natural environments.

Although based conceptually on the NADPH requirements of isoprene synthesis and the Farquhar photosynthesis model, our approach differs from that of Niinemets *et al.* (1999, 2004) in which isoprene production was assumed to be closely linked to the light-limited carbon assimilation rate (*A*_j_). This difference has important consequences, as we will show.

## TESTS OF THE HYPOTHESIS

We consider the observed environmental responses of isoprene emission (*Iso*) and also the ratio of isoprene emission to carbon gross assimilation (*Iso*/A_gross_), which is a sensitive indicator of the allocation of reducing power to the MEP pathway versus the Calvin cycle. We will also consider changes in the ratio of isoprene emission to carbon net assimilation (*Iso*/*A*_net_).

### Responses to PAR

**Fig. 2.**
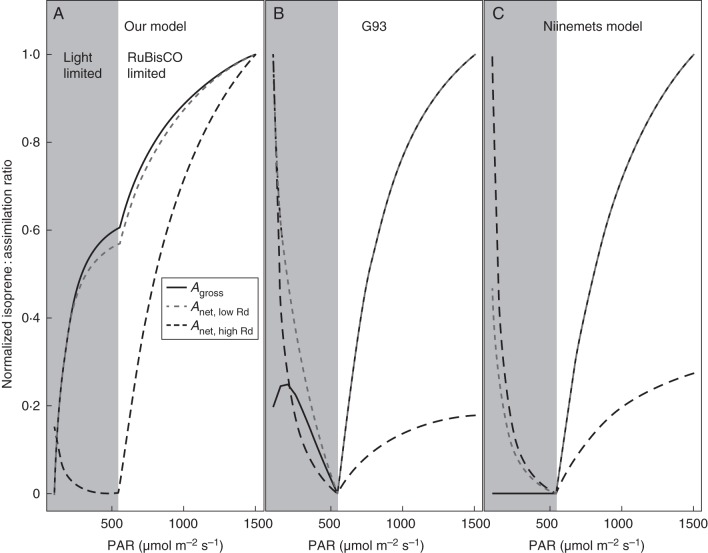
Modelled responses of the normalized ratio of isoprene to CO_2_ assimilation to changes in PAR for (A) our model, (B) G93 and (C) the Niinemets model. *T* = 30 °C, *c*_i_ = 273 µmol mol^−1^, *V*_cmax_25 °C_ = 70 µmol m^−2^ s^−1^, *J*_max_25 °C_ = 130 µmol m^−2^ s^−1^ based on values from Arneth *et al.* (2007*a*). The solid line represents the ratio of isoprene emission to gross assimilation, the dashed line to net assimilation. Normalized ratio of isoprene to CO_2_ net assimilation was simulated for two extreme values of dark respiration to illustrate the potential effect of the magnitude of *R*_d_ on how *Iso*/*A*_net_ varies with PAR: grey short-dashed line, low *R*_d;_ *R*_d_25 °C_ = 0·5 µmol m^−2^ s^−1^; black long-dashed line, high *R*_d;_ *R*_d_25 °C_ = 2 µmol m^−2^ s^−1^. Isoprene model parameters **a** and **b** (eqn 1) are based on data of Possell and Hewitt (2011; fig. 6).

Equation (1) predicts an increase of isoprene emission with PAR, but also an increase of the ratio *Iso*/*A*_gross_ (Fig. 2A). The predicted behaviour of *Iso*/*A*_net_ (*A*_net_ = *A* – *R*_d_, where *R*_d_ is mitochondrial respiration) is substantially different at low PAR, as shown in Fig. 2A. At saturating PAR, the difference becomes less important. This is due to the introduction of the *R*_d_ term, which affects the assimilation independently from the allocation of reducing power between carbon fixation and secondary metabolism. Most laboratory experiments have reported only *A*_net_; this should be kept in mind while interpreting the results.

The response of normalized *Iso*/*A*_gross_ (and *Iso*/*A*_net_) with PAR is predicted to take place in three stages (Fig. 2A).

#### Stage 1: light-limited carbon assimilation

This stage occurs when PAR absorbed is insufficient to generate an electron flux to satisfy Rubisco capacity. It is characterized by an initial steep increase of *Iso*/*A*_gross_ with PAR, becoming gradually less steep at higher PAR. For *Iso*/*A*_net_ the form of the response at low PAR depends on the magnitude of *R*_d_.

#### Stage 2: transition between light- and Rubisco-limited carbon assimilation

This stage is characterized by a discontinuity (abrupt increase) in the slope of *Iso*/*A*_gross_ (and *Iso*/*A*_net_) versus PAR.

#### Stage 3: Rubisco-limited carbon assimilation

When the electron requirement for carbon assimilation is fully satisfied, the additional reducing power generated by increasing PAR allows *Iso*/*A*_gross_ to continue increasing while *A*_gross_ remains constant. In this stage, *Iso*/*A*_net_ follows a similar pattern of the *Iso*/*A*_gross_ and increases with PAR. With still further increases in PAR, *Iso*/*A*_gross_ and *Iso*/*A*_net_ eventually saturate, as *J* tends to its maximal value (*J*_max_).

Note that the PAR flux where the transition between light- and Rubisco-limited assimilation occurs (Stage 2) as well as the rate of increase of *Iso*/*A*_gross_ with increasing PAR are dependent on both the photosynthetic and the isoprene model parameters.

We also examined the normalized responses of *Iso*/*A*_gross_ and *Iso*/*A*_net_ to changes in PAR in the G93 and Niinemets models (Fig. 2B, C). Under light-limited conditions (Stage 1), the picture differs dramatically depending on the model. In the Niinemets model, isoprene emissions are tightly linked to *A*_j_ (see Supplementary Data) and therefore the response of *Iso* to PAR necessarily has the same shape as that of *A*_j_, irrespective of the chosen values of *V*_cmax_ or *J*_max_. As a result, the ratio *Iso*/*A*_gross_ in this model is always constant under light-limited conditions, where carbon assimilation is equal to *A*_j_. The ratio *Iso*/*A*_net_, when simulated with the Niinemets model, always decreases with PAR under light-limited conditions. In G93, by contrast, changes in *Iso*/*A*_gross_ with PAR are strongly dependent on *V*_cmax_, *J*_max_ and temperature under light-limited conditions. Consequently, the increase followed by a decrease of *Iso*/*A*_gross_ under light-limited conditions, shown in Fig. 2B, is one of the possible responses of *Iso*/*A*_gross_ for G93, obtained for the temperature and photosynthetic parameters chosen for this simulation. Changing those parameters changes the shape of the response, and *Iso*/*A*_gross_ can decrease or increase at low PAR. Introducing a dark respiration term affects the shape of the response of *Iso*/*A*_net_ with PAR, as represented by the dashed lines in Fig. 2B. Hence, G93 can potentially show an *Iso*/*A* response to PAR similar to that of our model.

All three models predict increasing *Iso*/*A*_gross_ with PAR under Rubisco-limited conditions. Indeed, in the Niinemets model, as isoprene emissions are linked to *A*_j_, they must continue to increase even when carbon assimilation is Rubisco-limited. In that sense, the Niinemets model implicitly allows consumption of extra NADPH above the needs for carbon assimilation (for the PAR response only). For G93, isoprene emission approaches an asymptotic value at high PAR, while the Farquhar model fully saturates under Rubisco-limited conditions at high PAR.

Most studies reporting the fraction of assimilated carbon that is re-emitted as isoprene have found that it increases with PAR, in line with our predictions (Sharkey and Loreto, 1993; Harley *et al.*, 1996; Lerdau and Keller, 1997; Niinemets *et al.*, 2010). However, one study (Lerdau and Throop, 1999) found no significant increase in *Iso*/*A*_net_ with PAR for most of the tropical taxa they investigated.

**Fig. 3.**
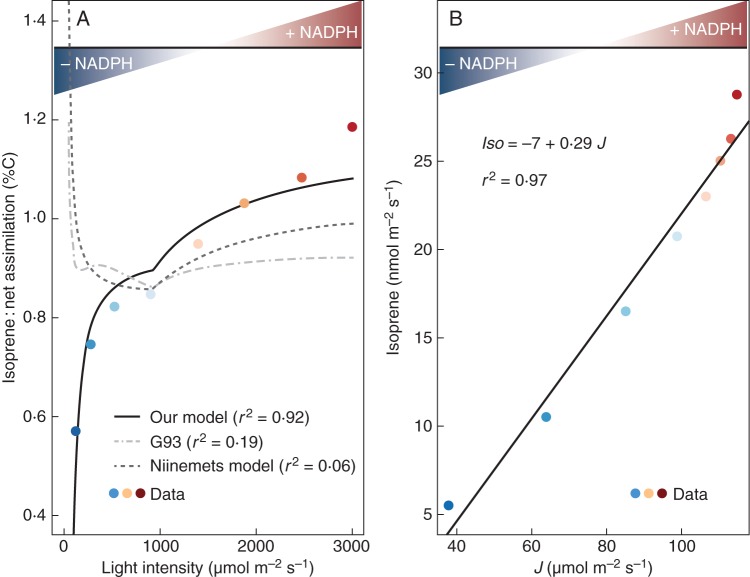
The relationship between isoprene emission and NADPH availability for carbon assimilation with changing PAR. (A) Increasing values of the isoprene to CO_2_ net assimilation ratio with increasing PAR, based on data digitized from figure 2 in Sharkey and Loreto (1993). Simulations made with our model, G93, and the Niinemets model are as indicated in the key. (B) The linear regression between isoprene data and the light-limited electron flux (*J*). Plant-specific isoprene parameters (**a**, **b**) are estimated from this linear regression and parameters for assimilation (*V*_cmax_, *J*_max_) were fitted to the assimilation observations by minimizing the residual sum of squares (RSS). In both panels, the availability of reducing power (NADPH) for CO_2_ assimilation is illustrated by a colour scheme, from dark blue (deficit) to dark red (excess).

Figure 3A compares the relationships of *Iso*/*A*_net_ to PAR in our model and in digitized data from Sharkey and Loreto (1993) on kudzu leaves (*Pueraria lobata*). Assuming *J*_v_ is constant (no variation in *c*_i_; Fig. 3B), the observed isoprene emissions show a strong positive linear relationship with *J* (*r*^2^ = 0·97). The model parameters **a** and **b** (eqn 1) have been estimated from this linear regression. The Farquhar model parameters were estimated with a best data/model fit by minimizing the residual sum of squares (RSS). The comparison between our model and the data for *Iso*/A_net_ shows excellent agreement (*r*^2^ = 0·92). In comparison, G93 and the Niinemets model both show poor agreement (*r*^2^ = 0·19 and *r*^2^ = 0·06, respectively). Yet the three models show a good agreement of the modelled isoprene alone (*Iso*) with data (our model: *r*^2^ = 0·97; G93: *r*^2^ = 0·92; Niinemets: *r*^2^ = 0·97; results not shown).

**Fig. 4.**
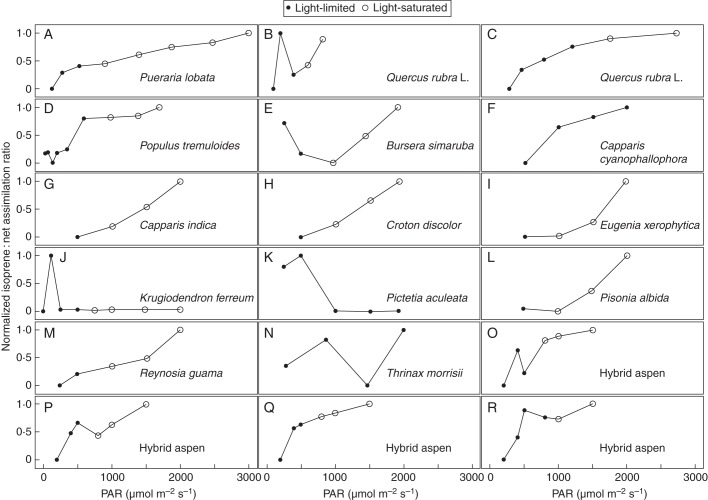
Observed responses of the normalized ratio (isoprene emission/net CO_2_ assimilation) to changes in PAR. Light-limited and light-saturated carbon assimilation as indicated in the key. Data digitized from (A) Sharkey and Loreto (1993), (B, C) Loreto and Sharkey (1990), (D) Monson and Fall (1989), (E–N) Lerdau and Keller (1997), (O–R) Sun *et al.* (2012). (O) Plant grown at ambient CO_2_, chamber [CO_2_] = 380 µmol mol^−1^. (P) Plant grown at ambient CO_2_, chamber [CO_2_] = 780 µmol mol^−1^. (Q) Plant grown at elevated CO_2_, chamber [CO_2_] = 380 µmol mol^−1^. (R) Plant grown at elevated CO_2_, chamber [CO_2_] = 780 µmol mol^−1^.

We also compiled data on the response of *Iso*/*A*_net_ to PAR from the limited number of published studies to assess the generality of the pattern (Fig. 4). The publications reported *A*_net_ rather than *A*_gross_, and did not typically provide measurements of *R*_d_. As the predicted response of *Iso*/*A*_net_ for low PAR is dependent on *R*_d_, it is not surprising to observe an initial decline in *Iso*/A_net_ with PAR for some of the 18 experiments. More importantly, the great majority of the studies show increasing *Iso*/A_net_ up to the highest PAR fluxes, especially when photosynthesis saturates (open circles in Fig. 4). In some studies a drop in assimilation rate at high PAR contributed to this increase in *Iso*/A_net_ at high PAR; this was probably due to stomatal closure at high PAR, resulting in reduced *c*_i_.

As shown in Fig. 2, the Niinemets model cannot reproduce the positive response of *Iso*/A_net_ to PAR that is generally observed under low PAR. Our model, along with G93, fully captures the shape of the observed response of *Iso*/A_net_ to PAR over the full range of PAR. But our model also provides a process-based explanation for this response.

**Fig. 5.**
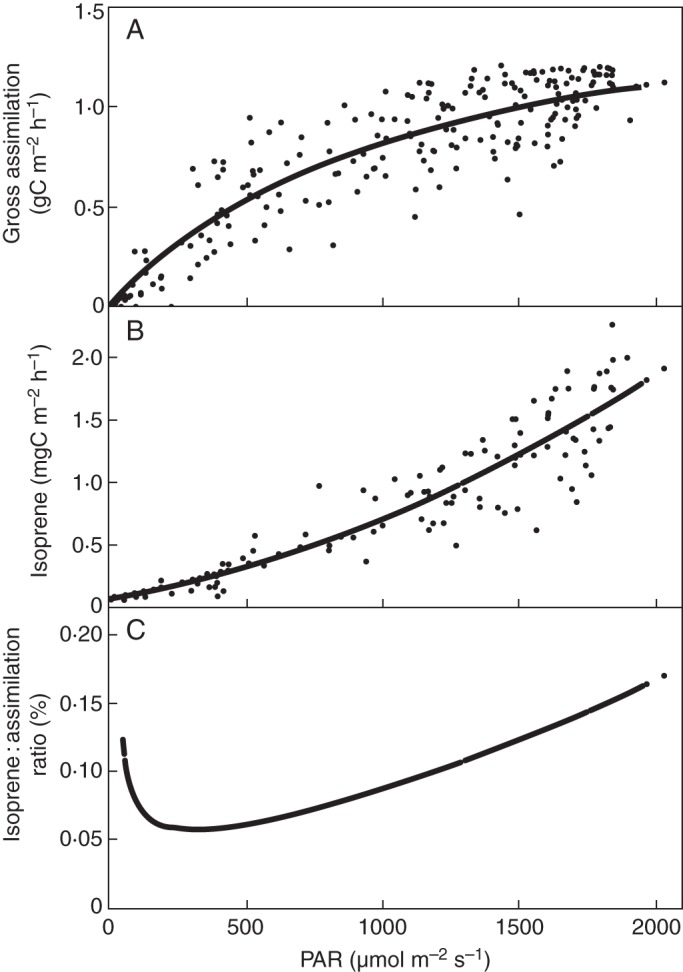
Above-canopy gross assimilation (A), isoprene emissions (B) and isoprene emission/gross assimilation (C), in relation to PAR. From flux measurements at Harvard Forest.

We also examined the relationship between *Iso*/*A*_gross_ and PAR at the canopy scale, at which isoprene emission is more likely to be controlled by the DMADP pool size than by isoprene synthase activity (Vickers *et al.*, 2010). We used simultaneous CO_2_ and isoprene flux measurements made at Harvard Forest, Massachusetts, USA (42·54°N, 72·17°W) (Urbanski *et al.*, 2007; McKinney *et al.*, 2011). Data used were obtained during the 2007 growing seasons using eddy covariance, with proton transfer reaction mass spectrometry used to measure the isoprene mixing ratio (McKinney *et al.*, 2011). Daytime data were selected for temperatures above 23 °C where variations in isoprene emission were no longer significantly driven by temperature (Fig. A1). Ecosystem respiration, estimated from night-time CO_2_ flux measurements, was used to convert the daytime measured net ecosystem CO_2_ exchanges into canopy-scale gross assimilation rates. Canopy-scale carbon assimilation shows a typical rectangular hyperbolic response to PAR, but the response of isoprene emission to PAR is closer to linearity, and emissions do not saturate at high PAR (Fig. 5A, B). Thus, above a PAR threshold of approx. 300 µmol m^−2^ s^−1^, *Iso*/A_gross_ increases with PAR even at high PAR, where assimilation is light-saturated, consistently with our hypothesis.

Scaling from leaf to canopy involves additional processes, such as within-canopy chemistry and canopy structure effects (Grote, 2007; Keenan *et al.*, 2011; Bryan *et al.*, 2012). Therefore, a canopy model is needed to fully account for these results, especially for low PAR where deposition processes can influence the observed above-canopy isoprene emissions and possibly explain the observed drop in *Iso*/*A*_gross_. Nevertheless these results, along with those of laboratory experiments, corroborate the notion that isoprene emission is related to the availability of electrons generated in photosynthesis, relative to the demand for them to be used in carbon assimilation.

### Responses to *c*_i_

Responses of isoprene emission to ambient CO_2_ concentration have been widely reported. Plants grown at high atmospheric CO_2_ concentrations generally emit less isoprene than those grown at lower CO_2_ concentrations. On short time scales, isoprene emission has also been shown to respond strongly and rapidly to *c*_i_, with lower emission rates at higher *c*_i_ (Rosenstiel *et al.*, 2003; Wilkinson *et al.*, 2009; Possell and Hewitt, 2011; Sun *et al.*, 2012). The fact that rapid changes in *c*_i_ evoke instantaneous responses in isoprene emission suggests that the driving mechanism must be tightly linked to processes in the chloroplast.

The mechanisms behind the decoupling between isoprene emission and carbon assimilation in the response to *c*_i_ are not well established. Niinemets *et al.* (1999) hypothesized that the dependency of isoprene emission on *c*_i_ might be due to the partitioning of reducing power and ATP into the MEP pathway. However, the model of Niinemets *et al.* (1999) does not allow for any greater partitioning of reducing power to the MEP pathway at low *c*_i_. Isotopic labelling studies have provided evidence for the existence of extra-chloroplastic sources of carbon to support isoprene production. Hence, competition for phosphoenolpyruvate (PEP) between cytosolic and chloroplastic processes has been proposed as an explanation for the drop in isoprene emission at high *c*_i_ due to the CO_2_-dependence of PEP carboxylase activity (Karl *et al.*, 2002; Rosenstiel *et al.*, 2003; Possell and Hewitt, 2011; Trowbridge *et al.*, 2012). But these experiments compared plants grown at different CO_2_ concentrations. Gene expression involving changes in enzyme quantities cannot explain the observed fast (about 10-min) responses to changes in *c*_i_. We focus here only on the short-term responses to *c*_i_, in which isoprene emission appears to be tightly coupled to changes in the pool size of DMADP (Rasulov *et al.*, 2009). Specifically, we examine whether the fast responses of isoprene emission to *c*_i_ could be explained in a simple way by our model, based on the same mechanisms we have proposed to explain the response to PAR.

**Fig. 6.**
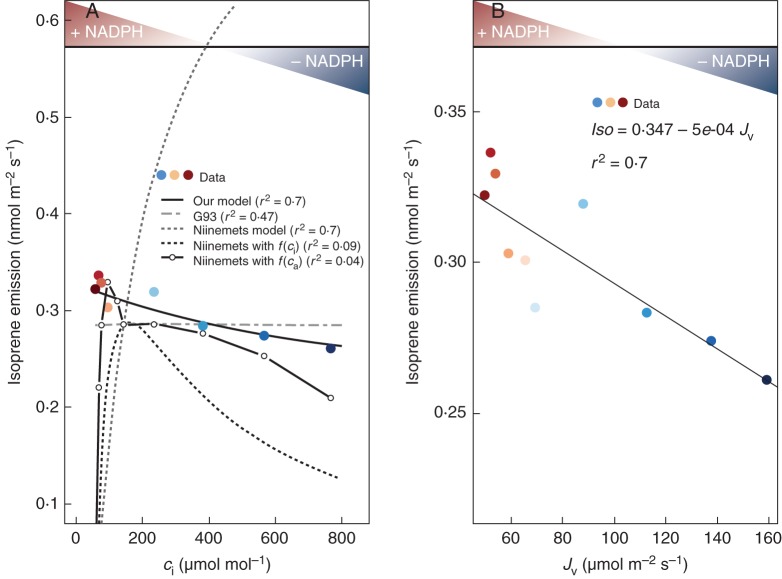
The relationship between isoprene emission and NADPH availability for carbon assimilation with changing internal CO_2_ concentration *c*_i_. (A) Decreasing isoprene emissions with increasing leaf-internal CO_2_ concentration, *c*_i_ (data from Possell and Hewitt, 2011); *T* = 30 °C, PAR = 1000 µmol m^−2^ s^−1^. Simulations made with our model, G93 and the Niinemets model are as indicated in the key. The dashed black line represent the Niinemets model with an additional CO_2_ effect represented by *f*(*c*_i_)= *c*_i_ [*c*_a_ = 390 µmol mol^−1^]/*c*_i_, where *c*_a_ is the atmospheric CO_2_ concentration. The plain grey line represent the Niinemets model with an alternative additional CO_2_ effect represented by *f*(*c*_a_)= [*c*_a_ = 390 µmol mol^−1^]/*c*_a_. The terms *f*(*c*_i_) and *f*(*c*_a_) are adapted from Arneth *et al.* (2007*a*). Standard isoprene emission factor (*I*_s_) is taken as the observed emission at *c*_a_ = 390 µmol mol^−1^. (B) The linear regression between isoprene data and the electron flux required for carbon assimilation by Rubisco (*J*_v_). Plant-specific isoprene parameters (**a**, **b**) are estimated from this linear regression and parameters for assimilation (*V*_cmax_, *J*_max_) were fitted to the observations by minimizing the residual sum of squares (RSS). In both panels, the availability of reducing power (NADPH) for CO_2_ assimilation is represented by a colour scheme, from dark blue (deficit) to dark red (excess).

**Fig. 7.**
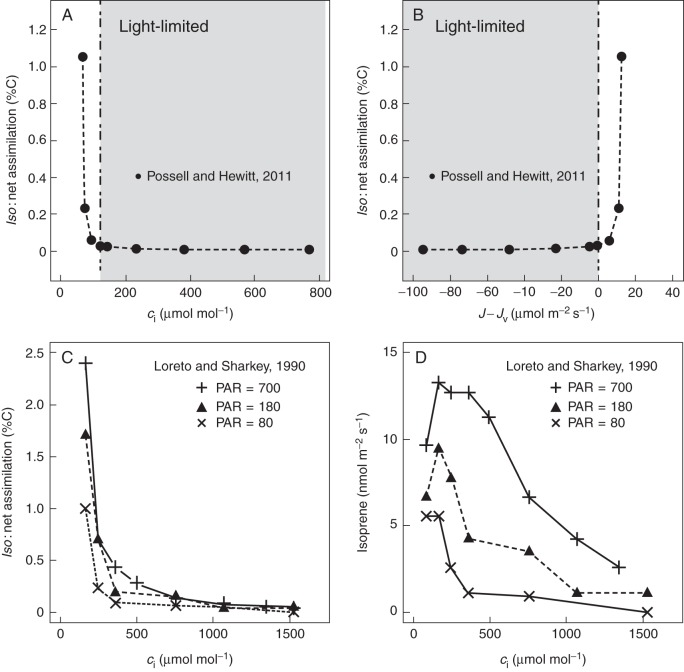
Observed changes in the ratio of isoprene emission to net carbon assimilation with changes in (A) leaf-internal CO_2_ concentration (*c*_i_), (B) electron excess (*J – J*_v_) (data from Possell and Hewitt, 2011). Observed changes with *c*_i_ of (C) the ratio of isoprene emission to carbon assimilation and (D) isoprene emission, for three PAR fluxes (μmol m^−2^ s^−1^). Data digitized from Loreto and Sharkey (1990).

At low *c*_i_, carbon fixation is Rubisco-limited, resulting in an excess of NADPH (Figs 1 and 6A). The excess of NADPH can be smaller or larger depending on PAR. This provides a simple explanation for why isoprene responses to changes in *c*_i_ are light-dependent (Loreto and Sharkey, 1993; Fig. 7D). Moreover, our model can indeed reproduce the isoprene emission response to changes in *c*_i_. This is shown in Fig. 6A using data on *Acacia nigrescens* from Possell and Hewitt (2011). Here, isoprene emission shows a strong negative linear relationship with the Rubisco-limited electron flux, *J*_v_ (*r*^2^ = 0·70), as shown in Fig. 6B. The parameters **a** and **b** (eqn 1) of our model were estimated from this linear regression. When plotted against *c*_i_, our model shows a good agreement with the data (*r*^2^ = 0·70). Figure 6A also shows the response of the G93 and the Niinemets model with and without a CO_2_ inhibition effect (Arneth *et al.*, 2007*a*; Pacifico *et al.*, 2011). It is clear from Fig. 6A that these models do not reproduce the observed response of isoprene emission to *c*_i_. Without an additional empirical function for CO_2_ inhibition, isoprene emissions simulated with the Niinemets model are quite out of range. Instead, the model shows a strong negative correlation with the data (*r*^2^ = 0·7). The negative relationship can be explained by the fact that although the PAR and therefore the light-limited electron flux (*J*) are constant, light-limited assimilation (*A*_j_) is strongly *c*_i_-dependent. Adding an empirical function to represent the CO_2_ inhibition effect, as in Arneth *et al.* (2007*a*), changes the shape of the response (allowing a decrease at high *c*_i_) but still the simulated emissions agree poorly with the data.

Again using the data from Possell and Hewitt (2011), we plotted *Iso*/*A*_net_ versus *c*_i_ (Fig. 7A) and *J* – *J*_v_ (Fig. 7B). These plots confirm that the fraction of assimilated carbon allocated to isoprene production increases under conditions of NADPH excess. This provides a simple explanation for the response of isoprene emission to *c*_i_. The extremely steep rise in *Iso*/*A*_net_ when *J* – *J*_v_ becomes positive is due to the combination of steeply increasing isoprene emission with decreasing assimilation rate as *c*_i_ declines.

**Fig. 8.**
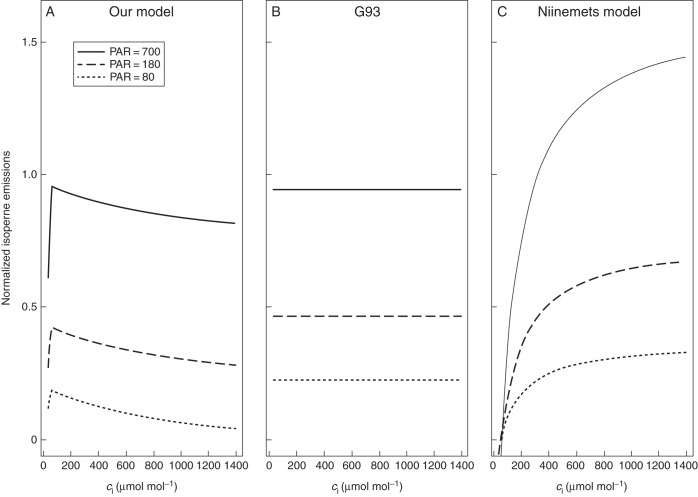
Modelled responses of isoprene emission versus *c*_i_ for three PAR fluxes (80, 180 and 700 µmol m^−2^ s^−1^). (A) our model, (B) G93 and (C) the Niinemets model. Parameters values as in Fig. 2. Emissions were normalized to a standard emission rate at *T* = 30 °C, *c*_i_ = 273 µmol mol^−1^, PAR = 1000 µmol m^−2^ s^−1^.

Loreto and Sharkey (1990) measured changes in isoprene emission with changing *c*_i_ at different PAR fluxes in *Quercus rubra*. Both *Iso*/*A*_net_ and isoprene emission are shown (Fig. 7C, D) to increase with PAR, consistent with a dependence on NADPH availability, at all values of *c*_i._ We compared the responses of G93 and the Niinemets model to *c*_i_ at different PAR fluxes, together with our model (Fig. 8). Note that both G93 and the Niinemets model are applied here in their original formulations (see Supplementary Data for details), and therefore do not include additional parameterizations of the CO_2_ effect. A number of studies have used these same models with additional empirical functions, introduced specifically to account for the observed CO_2_ inhibition (Arneth *et al.*, 2007*b*; Heald *et al.*, 2009; Pacifico *et al.*, 2011). G93 in its original formulation simulates no change at all in isoprene emission with changes in *c*_i_, although it has isoprene emission depending on PAR (Fig. 8B). The Niinemets model in its original formulation also simulates increasing isoprene emission with PAR, but here the modelled emissions *increase* with increasing *c*_i_, due to the fact that this model tightly links isoprene emission to light-limited assimilation (Fig. 8C). Thus, additional functions are required in both models to account for the observed effects of varying *c*_i_. In contrast, our model (Fig. 8A) can reproduce the form of the *c*_i_ response shown in the data (Fig. 7D), as well as the effects of combined changes in *c*_i_ and PAR (Fig. 7D), without the need for any additional function.

### Responses to leaf temperature

The temperature dependence of isoprene emission differs from that of photosynthesis. Temperature optima for carbon assimilation are usually ≤ 30 °C in C_3_ plants, while isoprene emission peaks at ≈ 40 °C (Guenther *et al.*, 1993; Niinemets *et al.*, 1999; Sharkey and Yeh, 2001; Pacifico *et al.*, 2009). An increase of *Iso*/*A* with temperature is usually observed (Sharkey and Loreto, 1993; Harley *et al.*, 1996; Sharkey *et al.*, 1996; Niinemets *et al.*, 1999; Sharkey and Yeh, 2001). The optimum for isoprene emissions rarely exceeds 40 °C, so the temperature dependence of isoprene emission cannot be fully explained by the temperature dependence of isoprene synthase, which is maximally active between 45 and 48 °C (Monson *et al.*, 1992; Lehning *et al.*, 1999; Niinemets *et al.*, 1999; Rasulov *et al.*, 2010). The decrease in isoprene emissions above 40 °C has long been considered to be linked to the behaviour of the photosynthetic electron transport rate (Guenther *et al.*, 1991; Niinemets *et al.*, 1999). Rasulov *et al.* (2010) found that this decrease is associated with decline in the DMADP pool size and the energetic status of the leaf.

In G93 the temperature dependency of isoprene emission is fixed with a temperature optimum around 38 °C. In the Niinemets model it is assumed to be primarily controlled by IspS activity, with the fraction of electrons used for isoprene production exponentially increasing with temperature. The temperature optimum for isoprene emissions in the Niinemets model is thus close to the optimum for IspS. Some global-scale studies have set an upper limit for the increase of ɛ with temperature, thereby reducing the temperature optimum to a value closer to 38 °C (Pacifico *et al.*, 2011) (Supplementary Data A.2).

**Fig. 9.**
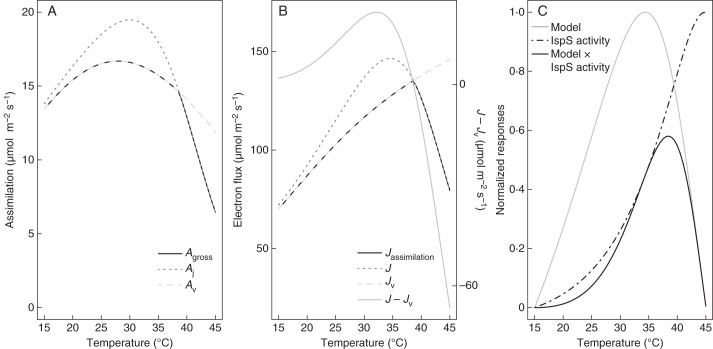
Explanation of the predicted temperature dependency of isoprene emissions. (A) The responses to temperature of the light-limited *A*_j_, the Rubisco-limited *A*_v_ and the gross assimilation *A*_gross_ (as indicated in key); (B) the associated electrons fluxes (left-hand axis) and the associated electron availability (*J – J*_v_) (right hand axis). (C) The normalized responses to temperature of our model, normalized IspS activity and the resulting product (see key). Temperature dependency of IspS is as described in Niinemets *et al.* (1999). Parameters values of the model are taken as in Fig. 2.

**Fig. 10.**
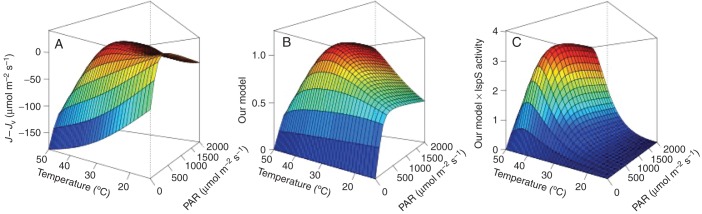
Responses to variation in temperature (°C) and PAR (μmol m^−2^ s^−1^) of electron availability (*J – J*_v_) (μmol m^−2^ s^−1^) (A), our model (B) and our model simulations multiplied by a normalized function of enzymatic activity (C). Farquhar model parameters are for *Quercus robur*, as described in Medlyn *et al.* (2002). Isoprene model parameters **a** and **b** (eqn 1) are based on data of Possell and Hewitt (2011) (fig. 6). Model outputs in A and C are normalized to be unity at *T* = 30 °C and PAR = 1000 µmol m^−2^ s^−1^.

Our model is based on the hypothesis that the production of DMADP depends on photosynthetic electron flux and variations in electron availability for functions other than carbon assimilation. Thus, our modelled optima for isoprene emissions are primarily driven by the behaviour of the light-limited electron flux. Figures 9 and 10 illustrate how a temperature response arises in our model. Carbon assimilation follows the lower of the temperature response curves of the Rubisco and light-limited assimilation rates. Rubisco activity usually has a higher temperature optimum than electron transport (Crafts-Brandner and Salvucci, 2000; Medlyn *et al.*, 2002; Cen and Sage, 2005; Kattge and Knorr, 2007). At high PAR an excess of NADPH can arise for temperatures below the optimum for electron transport (*J*), so isoprene emissions increase. Above this optimum (*T*_opt_J_), *J*_v_ still increases even if assimilation is reduced, due to the higher affinity of Rubisco for O_2_ at high temperatures. Both *J* and (*J* − *J*_v_) decrease for temperatures higher than *T*_opt_J_ (as illustrated in Fig. 9B). Our model thereby predicts a temperature optimum of isoprene emissions that is closer to the temperature optimum of the electron transport rate. Beyond this optimum, our model predicts a drop in the availability of reducing power, leading to a decrease of DMADP and consequently isoprene emissions. At low PAR (light-limited condition), however, (*J* − *J*_v_) decreases with increasing temperature, compensating for the increase of *J*. Predicted emissions are thus almost insensitive to temperature or even decrease with temperature (Fig. 10A, B). This behaviour is not realistic, so the model may be overestimating the effect of (*J* − *J*_v_ ) at low PAR.

We infer that energetic control alone is insufficient to fully explain the observed temperature dependency of isoprene emission. In principle the activities of enzymes along the MEP pathway should also influence the production rate of DMADP, but very little is known about their temperature responses (Zimmer *et al.*, 2000). Temperature optima for isoprene production are shifted toward higher temperature than *T*_opt_J_, probably because a decrease in DMADP pool size is compensated for by an increase in IspS activity (Rasulov *et al.*, 2010). Taking into account the temperature response of IspS, we can reproduce this shift (Figs 9C and 10C). So we suggest that temperature effects on enzyme activity may need to be considered, as well as temperature effects on electron availability.

A further limitation of our model is the paucity of available information on the temperature responses of *J*_max_ and *V*_cmax_ (Medlyn *et al.*, 2002; Kattge and Knorr, 2007). The experiments needed to quantify these responses are time-consuming, and in particular, few studies have included temperatures >40 °C. In general we would expect a decline in DMADP production for temperatures >40 °C due to thylakoid damage, while at temperatures above 45–48 °C irreversible damage to enzyme function will cause isoprene emission to cease.

**Fig. 11.**
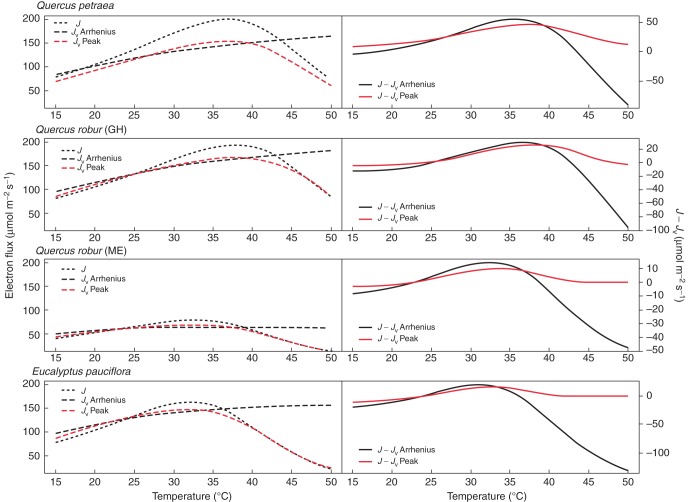
Left: temperature responses for different species of the light-limited electron flux (*J*) (dark grey dotted line), the Rubisco-limited electron flux (*J*_v_) using an Arrhenius function for *V*_cmax_ (*J*_v_ Arrhenius, black dashed line), and the Rubisco-limited electron flux using a peak function for *V*_cmax_ (*J*_v_ peak, red dashed line). Right: the resulting temperature responses of (*J – J*_v_), using an Arrhenius function for *V*_cmax_ (black solid line), and a peak function for *V*_cmax_ (red solid line). Farquhar parameters and calculation of *V*_cmax_ are as described in Medlyn *et al.* (2000). For *Quercus robur*: GH, greenhouse experiment; ME, mini-ecosystem experiment (Medlyn *et al.*, 2000). Simulations are done for *c*_i_ = 273 µmol mol^−1^ and PAR = 1000 µmol m^−2^ s^−1^.

Using data from Medlyn *et al.* (2000) and references therein, we checked variations with temperature of electron availability among isoprene emitting species at 1000 µmol m^−2^ s^−1^ PAR (Fig. 11). We also tested the influence of the temperature response parameterization of *V*_cmax_ by contrasting an Arrhenius function with a peak function (Supplementary Data), as described in Medlyn *et al.* (2000). The temperature optima for the selected species are all higher for *V*_cmax_ than *J*_max_ (Medlyn *et al.*, 2002; Kattge and Knorr, 2007). Consequently, we predicted a decline in DMADP pool size above *T*_opt_J_, due to the decline of *J* being accompanied by a decline in (*J* − *J*_v_), but the shape of the decline depended on the parameterization adopted.

## DISCUSSION

We have used a conceptual model to ask whether variation in the availability of NADPH in the chloroplast can plausibly account for observed changes in isoprene emission with PAR, *c*_i_ and leaf temperature. The answer is yes. By modelling isoprene emission as proportional to a simple metric of the excess or deficit of electrons relative to the demands of carbon assimilation, we have provided a unifying explanation for the lack of close coupling of isoprene emission with carbon assimilation, the disparities in carbon allocated to isoprene production, high isoprene emissions at low *c*_i_ and the shift of the temperature optimum for isoprene emission above that of carbon assimilation but below that of isoprene synthase.

To our knowledge, this is the first study that has attempted to model the flux of reducing power into the MEP production pathway based on the idea of a balance between electron supply and demand. Our hypothesis invokes mechanisms that are incompletely understood and thus is to some extent speculative. Nevertheless, it appears to have significant predictive power in explaining the already documented responses of isoprene emission to PAR, *c*_i_ and (with some caveats) temperature. Moreover, this hypothesis provides a parsimonious explanation for the response of isoprene emission to drought. Under moderate to mild drought where the photosynthetic apparatus is not damaged (Cornic and Briantais, 1991), carbon assimilation is first reduced by stomatal closure (and thus reduced *c*_i_). Under higher drought severity, this reduction is greatly increased by decreased ATP in water-deficient leaves (Lawlor and Tezara, 2009), which reduces the photosynthetic metabolic potential (*A*_pot_), even if *c*_i_ increases due to light respiration. The resulting oversupply of reducing power ensures that isoprene emissions continue at a high rate, although carbon assimilation is reduced (Niinemets, 2010). However, a decrease in ATP could also reduce isoprene emissions. Under extreme drought, however, damage to the photosynthesis apparatus eventually results in the cessation of both carbon assimilation and isoprene emission.

A strong diurnal cycle is observed in isoprene emission at canopy scales. Low emissions during early morning and late afternoon contrast with high emissions during the midday period (Hewitt *et al.*, 2011; Keenan and Niinemets, 2012). Our hypothesis explains this as a consequence of higher PAR and temperature, and lower *c*_i_ due to partial stomatal closure associated with higher evaporative demand in the midday period. This simple explanation does not require the intervention of a circadian clock, as had been proposed by Hewitt *et al.* (2011).

Note that we are *not* advocating a function of isoprene emissions as an ‘electron sink’ as was earlier proposed (e.g. Logan *et al.*, 2000). It is clear from the findings of Li and Sharkey (2012) that the quantity of electrons used in isoprene synthesis is far too small for this function to be plausible. The low affinity of IspS for DMADP already argues strongly against this notion (Silver and Fall, 1995; Schnitzler *et al.*, 1997). Our model implies that the allocation of reducing power to this pathway occurs under those conditions when electron availability is in excess, which fortuitously occurs during stress events when isoprene biosynthesis and emission is advantageous to the plant (e.g. Sharkey *et al.*, 2001; Vickers *et al.*, 2009).

Our results provide an alternative, robust approach to modelling isoprene emissions for global change applications. But more work is needed before implementing the model in a global context. Particular attention should be given to the influence of enzymatic activity on temperature responses of the modelled rates of isoprene. The values of the parameters **a** and **b** (eqn 1), and their potential species and environmental dependencies, also call for further investigation at several scales:

1. For leaves, by setting up experiments that could test interactions among the short-term responses of isoprene emission to different environmental drivers, and associated variations in the excess of electrons (i.e. isoprene/assimilation responses to *c*_i_ at different PAR fluxes, together with isoprene/assimilation response to PAR at different *c*_i_); and the influence of growth conditions on the parameters. Note that, as most of the process-based models are closely linked to photosynthesis models, information on the values of *V*_cmax_ and *J*_max_ associated with the isoprene standard emission rate would be valuable.
2. For ecosystems, by upscaling the model from the leaf scale to the canopy, with particular attention to the response of *Iso*/*A*_gross_. This step would require a representation of the canopy structure and vertical mixing as well as the canopy chemistry accounting for isoprene oxidation, deposition and OH regeneration.
3. At the global scale, with the possibility of using remotely sensed formaldehyde column concentrations to better constrain model parameters for different plant function types and environments. Formaldehyde is a product of isoprene oxidation. As it is observed by satellite, with global coverage, numerous studies have used formaldehyde data to investigate isoprene emission at larger scales (Palmer *et al.*, 2003, 2006; Barkley *et al.*, 2008; Stavrakou *et al.*, 2009; Fortems-Cheiney *et al.*, 2012).

A comprehensive approach to isoprene modelling would also have to account for longer-term acclimation over a time scale of weeks to months, including responses to antecedent temperatures (Guenther *et al.*, 2006), phenological stages, and differences between the short-term and acclimated responses to CO_2_ (Sun *et al.*, 2012), which are presumably mediated by transcriptional control of the MEP pathway enzymes. It would be of particular interest to examine whether these acclimatory changes in isoprene emission are correlated with acclimatory changes in reducing power. However, some acclimatory shifts are unlikely to be explained by a model based on reducing power alone. For example, growth at higher temperatures leads to increased emission rates measured at a common temperature (Pétron *et al.*, 2001; Niinemets *et al.*, 2010), whereas reducing power at a given temperature tends to be reduced by high growth temperatures due to a decline in the *J*_max_/*V*_cmax_ ratio (Hikosaka *et al.*, 1999; Onoda *et al.*, 2005). Longer-term responses of isoprene emission to changes in growth temperature are therefore presumably governed by other factors, including transcriptional control of the MEP pathway enzymes.

### Conclusions

A simple model of the biochemistry and physiology of isoprene emissions has been developed and used to test the hypothesis that the reducing power available to the synthesis pathway for isoprene varies according to demands of carbon assimilation. The model explains the observed response of isoprene production to environment and the coupling/decoupling between carbon assimilation and isoprene emission. The model has the potential to improve global-scale modelling of vegetation isoprene emissions, as well as emissions of isoprenoids that do not originate from storages.

## SUPPLEMENTARY DATA

Supplementary data are available online at www.aob.oxfordjournals.org and consist of details of the following. The G93 algorithm for prediction of isoprene emission from vegetation; the Niinemets model based on quantifying the NADPH cost for isoprene synthesis; and the model for photosynthetic carbon assimilation based on the Farquhar model.

Supplementary Data
