# Supplementary material for: A unifying conceptual model for the environmental responses of isoprene emissions from plants
Source: Ann Bot. 2013 Sep 19;112(7):1223–38. doi: 10.1093/aob/mct206 (PMC3806535; doi:10.1093/aob/mct206)
Supplement: Supplementary Data [file supp_112_7_1223__index.html]

A unifying conceptual model for the environmental responses of isoprene emissions from plants — A unifying conceptual model for the environmental responses of isoprene emissions from plants — Supplementary Data 

# A unifying conceptual model for the environmental responses of isoprene emissions from plants

## Supplementary Data

Supplementary Data

**Files in this Data Supplement:**

- Supplementary Data - Pdf file
