# Supplementary material for: A unifying conceptual model for the environmental responses of isoprene emissions from plants
Source: Ann Bot. 2013 Sep 19;112(7):1223–38. doi: 10.1093/aob/mct206 (PMC3806535; doi:10.1093/aob/mct206)
Supplement: Supplementary Data [file supp_mct206_mct206supp.pdf]

SUPPLEMENTARY DATA

A1. MEGAN - G93 (Guenther et al., 1993, 2006)

MEGAN is the most widely used model for prediction of isoprene emission from vegetation. This model was built on the Guenther et al. (1993) algorithm (G93) where the emission rate of isoprene (Iso) is calculated by multiplying a species-specific standard emission rate (I_S) by a set of empirical equations taking in account changes in environmental factors. The standard conditions for estimation of I_S are a leaf temperature of 30˚C and a PAR of 1000 μmol m^{-2} s^{-1}. For this study, we used the Guenther et al. (1993) algorithm (G93), where solely changes in PAR and temperature are taken in account:

$$Iso = I_S\ C_L\ C_T, \quad \textbf{(A1)}$$

with

$$C_L = \alpha\ C_{L1}\ PAR / \sqrt{(1+ \alpha^2\ PAR^2)} \quad \textbf{(A1a)}$$

$$C_T = \exp\ (\ C_{T1}\ (T - T_S) / (R\ T_S\ T)) / (\ C_{T3} + \exp\ (C_{T2}\ (T - T_m) / (R\ T_S\ T)) \quad \textbf{(A1b)}$$

T (K) is the leaf temperature, T_s is the temperature at standard conditions (= 303 K), R is the gas constant (= 8.314 J K^{-1} mol^{-1}), PAR is the photosynthetically active radiation. C_{L1}, α, C_{T1}, C_{T2}, C_{T3} and T_m are empirical coefficients:

C_{L1} = 1.066, α = 0.027, C_{T1} = 95 000 J mol^{-1}, C_{T2} = 230 000 J mol^{-1}, C_{T3} = 0.961 J mol^{-1} and T_m = 314 K.

A2. Niinemets model (Niinemets et al., 1999)

The Niinemets model is based on quantifying the NADPH cost for isoprene synthesis. It builds on the Farquhar model of photosynthesis.

The general concept is that a temperature-dependent fraction of the electron flux (ε) is used for isoprene production.

$\varepsilon = J_{iso} / J_{tot}$, **(A2)**

Where J_{iso} is the electrons flux required for a certain isoprene production (Iso) and J_{tot} is the total photosynthetic electron flux.

The total electron flux is approximated from the Farquhar model as follows:

$J_{tot} \approx J_{CO2+O2} = (A_{j\_net} + R_d)\ (4\ c_i + 8\ \Gamma^*)/(c_i - \Gamma^*)$, **(A2a)**

where A_{j_net} is the net light-limited carbon assimilation, R_d the dark respiration, c_i internal CO_2 concentration and Γ^* the CO_2 compensation point in the absence of dark respiration.

Given that one molecule of isoprene requires six carbon atoms and that the NADPH cost per molecule of carbon is 1.17 higher for isoprene production than for carbon assimilation, the electron flux needed for a given isoprene production Iso is calculated by:

$J_{iso} = 6\ \mathrm{Iso}\ 1.17\ (4\ c_i + 8\ \Gamma^*)/(c_i - \Gamma^*)$, **(A2b)**

Rearranging, we obtain:

$\mathrm{Iso} = \varepsilon\ J_{tot}\ (c_i - \Gamma^*)/\ (7.02\ (4\ c_i + 8\ \Gamma^*))$

$= \varepsilon / 7.02\ A_j$, **(A2c)**

where A_j is the gross assimilation under light-limited conditions.

The fraction of electrons allocated to isoprene production is calculated as from Arneth et al. (2007) and Pacifico et al. (2011):

$\varepsilon = \varepsilon_s\ \tau$, **(A2d)**

where ε_s is the fraction of electrons for isoprene production under the standard conditions of leaf temperature of T_s = 30˚C and PAR of 1000 μmol m^{-2} s^{-1} and $\tau = \min\{\exp[0.1\ (T - T_s)]\ ;\ 2.3\}$, is a function which mimics the enzymatic response reported by Niinemets et al. (1999).

A1. Model of photosynthetic carbon assimilation (Farquhar et al., 1980)

The model for photosynthetic carbon assimilation is based on the Farquhar model (Farquhar et al., 1980). Temperature responses of the different parameters are described in Medlyn et al. (2002), and based on previous work from (Harley et al., 1986; Long, 1991; Harley et al., 1992; Harley and Baldocchi, 1995; Lloyd et al., 1995; Bernacchi et al., 2001).

Calculation of the light limited electron flux (J):

$J = ((\alpha PAR + J_{max}) - \sqrt{((\alpha PAR + J_{max})^2 - 4\alpha\theta J_{max} PAR)})/(2\theta)$ **(A3a)**

$J_{max} = = J_{max25}.\exp(E_{aJ}\ (1/298.15 - 1/T)\ /R).\ J_1/J_2$ **(A3b)**

$J_1 = 1 + \exp((298.15\ \Delta S - E_{dJ})/R/298.15)$ **(A3c)**

$J_2 = 1 + \exp((T\ \Delta S - E_{dJ})/R/T)$ **(A3d)**

J_{max25} is the value of J_{max} at 25˚C, α = 0.385 mol electron mol^{-1} photon, θ is a curvature parameter of the light curve response and T is the temperature in K. Other symbols are summarized in Table A1.1

Calculation of the maximum Rubisco capacity V_{cmax}, Γ, Kc , Ko:*

$$V_{cmax} = V_{cmax25} \exp(E_a (1/298.15 - 1/T) /R) \quad \textbf{(A3e)}$$

$$K_c = 404.9 \exp(79430 (T - 298.15)/(298.15 RT)) \quad \textbf{(A3f)}$$

$$Ko = 278.4 \exp(36380 (T - 298.15)/(298.15 RT)) \quad \textbf{(A3g)}$$

$$\Gamma^* = 42.75 \exp(37830 (T - 298.15)/(298.15 RT)) \quad \textbf{(A3h)}$$

V_{cmax25} is the value of V_{cmax} at 25˚C, K_c and K_o are the Michaelis coefficients of Rubisco for CO_2 and O_2 respectively and Γ^* is the CO_2 compensation point in the absence of dark respiration. Other symbols are summarized in Table A1.1

Table A1.1 Description and values of the parameters of Farquhar model used in standard simulations (Medlyn et al., 2005)

Symbol	Definition	Unit	Value
E_{aJ}	Activation energy for J	kJ mol^{-1}	38.670
E_{dJ}	Desactivation energy for J	kJ mol^{-1}	200
E_a	Activation energy for V_{cmax}	kJ mol^{-1}	58. 520
E_d	Desactivation energy for V_{cmax}	kJ mol^{-1}	200
ΔS	Entropy term	kJ mol^{-1} K^{-1}	0.6381

Table A1.2 Values of the parameters of Farquhar and isoprene models for Fig. 3 and Fig.6

Symbol	Unit	Value (Fig.3)	Value (Fig.6)
J_{max25}	μmol m^{-2} s^{-1}	99.53	54.32
V_{cmax25}	μmol m^{-2} s^{-1}	62.8	56.9
a	unitless	$2.2\ 10^{-4}$	$4.86\ 10^{-5}$
b	unitless	$7\ 10^{-5}$	$5\ 10^{-6}$

Figure A1: Above canopy isoprene emissions in relation to air temperature for three ranges of PAR - Low (1000-1250 μmol m^{-2} s^{-1}), Medium (1250-1500 μmol m^{-2} s^{-1}) and High (1500-1750 μmol m^{-2} s^{-1}). Data from flux measurements at Harvard Forest.

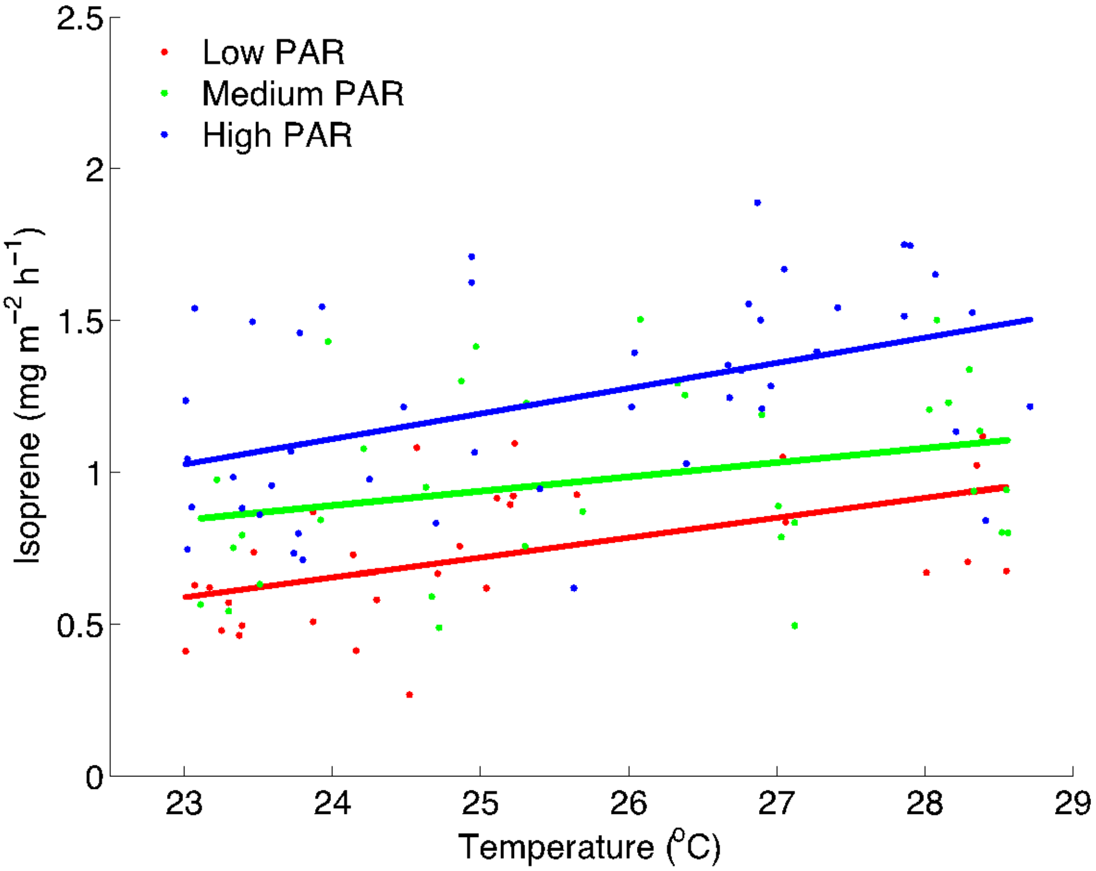

References

Arneth A, Niinemets Ü, Pressley S, Bäck J, Hari P, Karl T, Noe S, Prentice I, Serça D, Hickler T, Wolf A, Smith B. **2007**. Process-based estimates of terrestrial ecosystem isoprene emissions: incorporating the effects of a direct CO2-isoprene interaction. *Atmospheric Chemistry and Physics* **7**: 31–53.

Bernacchi CJ, Singsaas EL, Pimentel C, Portis ARJ, Long SP. **2001**. Improved temperature response functions for mod- els of Rubisco-limited photosynthesis. *Plant, Cell and Environment* **24**: 253–260.

Farquhar GD, Von Caemmerer S, Berry JA. **1980**. A Biochemical Model of Photosynthetic CO2 Assimilation in Leaves of C3 Species. *Planta* **149**: 78–90.

Guenther A, Karl T, Harley P, Wiedinmyer C, Palmer PI, Geron C. **2006**. Estimates of global terrestrial isoprene emissions using MEGAN (Model of Emissions of Gases and Aerosols from Nature). *Atmospheric Chemistry and Physics* **6**: 3181–3210.

Guenther AB, Zimmerman PR, Harley PC, Monson RK, Fall R. **1993**. Isoprene and Monoterpene Emission Rate Variability: Model Evaluations and Sensitivity Analyses. *Journal of Geophysical Research* **98**: 12609–12617.

Harley PC, Baldocchi D. **1995**. Scaling carbon dioxide and water vapour exchange from leaf to canopy in a deciduous forest. I. Leaf model parametrization. *Plant, Cell and Environment* **18**: 1146–1156.

Harley PC, Thomas RB, Reynolds JF, Strain BR. **1992**. Modelling photosynthesis of cotton grown in elevated CO2. *Plant, Cell and Environment* **15**: 271–282.

Lloyd J, Grace J, Miranda AC, Meir P, Wong SC, Miranda HS, Wright IR, Gash JHC, McIntyre J. **1995**. A simple calibrated model of Amazon rainforest productivity based on leaf biochemical properties. *Plant, Cell and Environment* **18**: 1129–1145.

Long SP. **1991**. Modification of the response of photosynthetic productivity to rising temperature by atmospheric CO2 concentrations: Has its importance been underestimated? *Plant, Cell and Environment* **14**: 729–739.

Medlyn BE, Dreyer E, Ellsworth D, Forstreuter M, Harley PC, Kirschbaum MUF, Roux XLE. **2002**. Temperature response of parameters of a biochemically based model of photosynthesis . II . A review of experimental data. *Plant, Cell and Environment* **25**: 1167–1179.

Medlyn BE, Berbigier P, Clement R, Grelle A, Loustau D, Linder S, Wingate L, Jarvis PG, Sigurdsson BD, McMurtrie RE. **2005**. Carbon balance of coniferous forests growing in contrasting climates: Model-based analysis. *Agricultural and Forest Meteorology* **131**: 97–124.

Niinemets Ü, Tenhunen JD, Harley PC, Steinbrecher R. **1999**. A model of isoprene emission based on energetic requirements for isoprene synthesis and leaf photosynthetic properties for Liquidambar and Quercus. *Plant, Cell and Environment* **22**: 1319–1335.

Pacifico F, Harrison SP, Jones CD, Arneth A, Sitch S, Weedon GP, Barkley MP, Palmer PI, Serça D, Potosnak M, Fu T-M, Goldstein A, Bai J, Schurgers G. **2011**. Evaluation of a photosynthesis-based biogenic isoprene emission scheme in JULES and simulation of isoprene emissions under present-day climate conditions. *Atmospheric Chemistry and Physics* **11**: 4371–4389.
